# A New Method for Evaluating the Diffusion of Ca^2+^ and OH^-^ Ions through Coronal Dentin into the Pulp

**Published:** 2012-10-13

**Authors:** Maria Giovanna Gandolfi

**Affiliations:** 1. Laboratory of Biomaterials and Oral Pathology, Department of Odontostomatological Sciences, University of Bologna, Bologna, Italy.

**Keywords:** Calcium, Calcium Hydroxide, Dentin, Diffusion, Dycal, Dental Materials, Endodontics, Pulp Cap, ProRoot, Mineral Trioxide Aggregate, Pulpdent Paste, Dental Pulp Exposure

## Abstract

**Introduction:**

Ca(OH)_2_-containing/forming materials are conventionally used for indirect pulp-capping and are theoretically able to release Ca^2+^ and OH^-^ ions for hydrolytic dissociation. However, no evidence exists for ion diffusion through the remaining coronal dentin. The aim of this study was to design an innovative experimental set-up to test the ability of Ca(OH)_2_-containing and Ca(OH)_2_-forming pulp-capping materials to generate pulpward Ca^2^ and OH^-^ ion fluxes through coronal dentin after indirect pulp-capping in vitro.

**Materials and Methods:**

Standardized class 1 cavities were prepared in erupted sound human molars. Pulp tissue was excised. A coronal Remaining Dentin Thickness (RTD) (1±0.2 mm thick) was prepared within an occlusal-to-pulp cavity system (coronal RD system). The whole system/sample was treated with 17% EDTA to remove the smear layer and the external surface was covered by nail varnish. Indirect pulp-capping was performed on coronal RDT using a conventional pulp-capping material covered by a glass ionomer cement, a composite and nail varnish. Chemically different Ca(OH)_2_ materials were used to test the reliability of the set-up. The leached Ca^2+^ and OH^-^ ions were measured using ion-selective electrodes after soaking for 3 hours, 24 hours, and 7 days in deionized water (10 mL, 37°C).

**Results:**

Calcium ions were detected and a rise in pH was observed in the treated water after a few hours for all tested materials.

**Conclusion:**

The experimental set-up proved to be an easy and effective method for testing the different Ca(OH)_2_-containing and Ca(OH)_2_-forming materials ability to induce a pulpward flux of calcium and hydroxyl ions through coronal remaining dentin after indirect pulp-capping. The new system will allow the screening of current pulp-capping materials.

## Introduction

Indirect pulp-capping is a procedure following caries removal in which a protective material is placed on a thin partition of remaining (carious/infected) dentin thickness (RDT) or slightly softened dentine [1][2].

Ca(OH)_2_-based/containing materials have been used for direct and indirect pulp-capping since 1940 [3]. They release free calcium (Ca^2+^) and hydroxyl (OH^-^) ions due to their ionic dissociation in the presence of fluids [4][5].

Calcium silicate materials, including Mineral Trioxide Aggregate (MTA) materials, are now conventionally used for pulp-capping [6] due to their ability to produce calcium hydroxide and release Ca^2+^ and OH^-^ ions during their hydration process [7][8][9][10][11]. Therefore, MTA cements can be considered Ca(OH)_2_-forming/releasing materials.

In the healing process, the biological properties of both Ca(OH)_2_-based and Ca(OH)_2_-producing materials for pulp-capping are related to their ion-releasing ability. Adequate concentrations of leached Ca^2+^ and OH^-^ ions can have strong biological affect on pulpal stem cells, on surrounding tissues and on infecting bacteria. Ca^2+^ ions induce the proliferation of human dental pulp cells and their differentiation into odontoblasts [12][13], the formation of a tertiary/reparative dentin bridge, the mineralization of dentin and the subtraction of environmental CO2 required for bacterial growth. OH^-^ ions (alkaline pH) have bacteriostatic and antibacterial/bactericidal activity (destruction of bacterial cytoplasmic membranes, protein denaturation, prevention of bacterial re-growth and re-entry into dentin tubules/pulp); they can neutralize of lactic acid produced by bacterial activity and promote the formation of apatite [14] as well as reparative dentin.

The present study aimed to develop a simple, reliable system to test the ability of calcium hydroxide materials to create a pulp ward flux of Ca^2+^ and OH^-^ ions through the remaining dentin thickness after indirect pulp-capping in vitro. To verify the reliability of the designed set-up, Ca(OH)_2_-containing and Ca(OH)_2_-producing materials with various different chemical compositions and characteristics were used: i) a non-setting aqueous calcium hydroxide (Pulpdent), ii) a self-setting sulfonamide- disalicylate calcium hydroxide (Dycal), and iii) a hydraulic water-based calcium silicate MTA cement (ProRoot MTA).

## Materials and Methods

### Coronal RDT system

Human caries-free erupted extracted third molars were sectioned at the cemento-enamel junction using a diamond saw.

**Figure 1:**
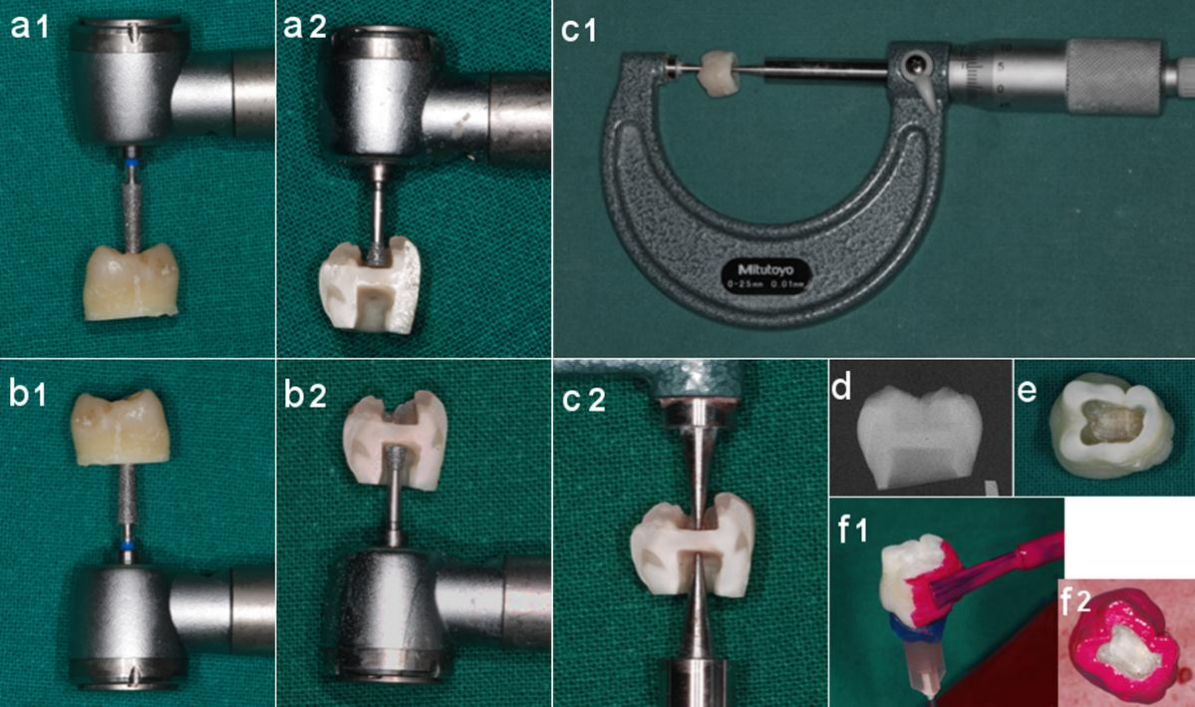
Coronal RDT system: a-c) Cavity preparation. Cross sections specifically prepared to show the coronal remaining dentin thickness (RDT) in the designed occlusal-to-pulp cavity system (coronal RDT system); c) The RDT was measured by a calliper); (d: Radiographic image of a whole coronal RDT system); e) Pulp-side of the dentin in a system); f) Nail varnish application

Standardized class 1 occlusal cavities were prepared on the coronal side (cavity-side chamber) using a high-speed, inverted-cone, flat-end diamond bur with a diameter of 0.2 mm (Figure 1a), (Figure 1b).

The pulp tissue was excised and the outermost pulp-side dentin was removed using the above-mentioned bur to obtain a standardized pulp-side chamber.

The remaining dentin thickness was standardized to 1±0.2 mm. The RDT was assessed using a caliper (Figure 1c) and by X-ray using a radiographic unit (Myray Cefla, Imola, Italy) (Figure 1d), and modified if necessary. Operative conditions for taking the X-ray were: 3 cm distance, 0.13 s exposure at 70 KVp and 8 mA. The film (Eastman Kodak, Rochester NY, USA) was processed (automatic developer, 4 min at 30°C) and scanned.

A sample definaed as “coronal RDT system” was obtained from each tooth (Figure 1e). Each sample was immersed into 4 mL of 17% EDTA (Ogna, Milan, Italy) for 3 minutes at room temperature to remove the smear layer. Samples were then thoroughly rinsed with deionized water.

Wet cotton pellets were put in both the coronal and pulpal cavities during the application of nail varnish to avoid dehydration of the dentin. The external/outer surface of the coronal RDT system (the enamel of the lateral surface and the dentin of the resected root) was covered by nail varnish to hamper the release of Ca^2+^ ions from the dental tissues, excepting the occlusal and the pulpal cavities (Figure 1f).

### Indirect pulp-capping

**Figure 2:**
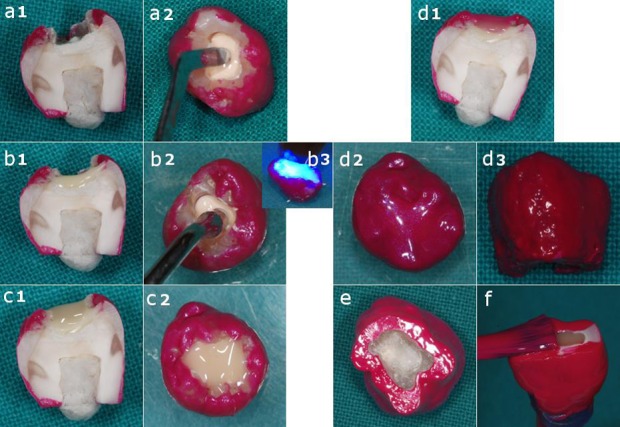
Cavity obturation. a) Pulp-capping material layered on the RDT. During the procedure, a wet cotton pellet was present in the pulp cavity); b) Vitrebond layered on the pulp-capping material and light-cured); c) Gradia composite layered on Vitrebond); d) Layer of nail varnish finally applied on the outer surface to isolate the obturation); d) Lateral side of the coronal RDT system covered by nail varnish); e) Lower side of a treated samples and positive controls. The pulp-side of the dentin is visible); f) Lower side of negative controls. The pulp cavity filled with Gradia is visible

Indirect pulp-capping on the RDT was performed using a conventional pulp-capping material (Figure 2a),(Figure 2b): either Pulpdent Paste Kit (Pulpdent Corp., Watertown, MA, USA) or Dycal (Dentsply, Caulk, Milford, DE, USA), as calcium hydroxide-containing materials, or white ProRoot MTA (Dentsply Tulsa Dental, Johnson City, TN, USA), as a calcium hydroxide-forming material.

The coronal obturations (n=3 for each group) were completed with a glass ionomer cement (Vitrebond, 3M, USA), a composite (Gradia, Direct Flo A2, GC Corp., Japan) and finally nail varnish (Figure 2c).

In positive controls the occlusal cavity was completely filled with Gradia and the whole surface of the system covered by nail varnish (Figure 2d), except the pulp cavity (Figure 2e).

In the negative controls both the occlusal and pulpal cavities (Figure 2f) were filled with Gradia (as a non-calcium releasing material) [15] and the external surface of the samples were entirely covered by nail varnish to hamper any ion release from the mineral tissue.

The samples were stored individually at a constant 37°C temperature in numbered polystyrene sealed containers (Kartell, Milan, Italy) filled with 10 mL of deionized water (Carlo Erba, Milan, Italy) [pH (25°C) 6.5±0.1] to create a simulated intrapulpal pressure equivalent to 3 cm H_2_O Sealed containers filled with 10 mL of deionized water and maintained at 37°C represented the control.

### Calcium and hydroxyl ions measurements

The Ca^2+^ and OH^-^ ions leached into the soaking water were measured using ion-selective electrodes after 3 hours, 24 hours and 7 days following placement of the samples.

Calcium ions (ppm) and hydroxyl ions (pH) were analyzed in the deionized water with a magnetic stirrer using a multiparameter laboratory meter (inoLab 750, WTW Weilheim, Germany) connected to a calcium probe (Calcium ion electrode, Eutech instruments Pte Ldt, Singapore) or a (selective) temperature compensated pH probe/electrode (SenTix Sur WTW, Weilheim, Germany). For calcium quantization, 0.200 mL (2%) of 4 mol/L KCl (ISA WTW, Weilheim, Germany) were added to 10 mL of deionized water.

Cumulative calcium release at each analysis time was obtained by adding the amount of calcium detected at that time to the amounts detected at all the previous measurements.

## Results

After immersion of the tooth samples for the various specific times, the treated water was found to be clear/limpid and no precipitates were present.

The amount of calcium ions (ppm) leached from the treated samples was observed for all the tested materials (Table 1) and (Table 2). The total released calcium was different from the various test materials (ProRoot MTA>Pulpdent>Dycal) but increased over time for all materials.

**Table 1:** Range of calcium ions released in soaking water (Ca^2+^ expressed as ppm, n=3)

** **	**3 hours**	**24 hours**	**7 days**
**Pulpdent**	1.3-2.4	1.3-3.1	3.6-5.44
**Dycal**	1.5-2.7	2.0-4.1	2.7-5.9
**ProRoot MTA**	2.1-3.0	2.8-4.6	4.0-8.4
**Negative control**	1.1-1.6	0.2-1.6	1.4-2.5
**Positive control**	1.2-1.8	0.3-2.1	1.8-2.9
**Deionized water**	0.4-0.9	0.2-0.8	0.6-0.9

**Table 2:** Cumulative calcium ions (ppm) released in soaking water (n=3)

** **	**3 hours**	**24 hours**	**7 days**
**Pulpdent**	1.8	4.2	8.0
**Dycal**	1.9	4.6	9.0
**ProRoot MTA**	2.6	6.0	12.5

The control deionized water showed traces of calcium; small amounts of calcium ions were also released by the negative and positive controls.

An alkaline pH was observed in the soaking water after 24 hours. The alkalinization of the treated water was first measured after 24 hours in soaking water (Pulpdent>Dycal>ProRoot MTA) and proved to increase in parallel with the length of soaking time for all the materials (Table 3). No differences in pH were observed in the soaking water after immersion of the negative controls compared to control deionized water. A slight increase in the pH of the soaking water was detected after immersion of the positive controls.

**Table 3:** Range of hydroxyl ions released in soaking water (OH^-^ expressed as pH, n=3)

** **	**3 hours**	**24 hours**	**7 days**
**Pulpdent**	6.3-7.2	7.1-8.2	7.4-7.9
**Dycal**	5.8-7.6	7.2-7.8	7.4-7.8
**ProRoot MTA**	6.2-7.3	7.1-7.5	7.5-7.8
**Negative control **	5.7-7.2	6.0-7.6	6.8-7.7
**Positive control**	6.4-7.0	6.8-7.5	6.7-7.4
**Deionized water**	6.4-7.4	6.9-7.2	6.8-7.5

## Discussion

This system evaluated in vitro the rate at which ions leached from pulp-capping materials and permeate through coronal dentin to the pulp chamber.

The ability of all the tested materials to generate a pulpward flux of biologically active ions demonstrated the suitability of the coronal RDT system. The data on calcium and hydroxyl ion diffusion detected for each treatment group at the same analysis times were relatively reproducible and showed limited dispersion.

No similar set-up has been designed and proposed to test pulp-capping materials and no prior studies on calcium ion diffusion through coronal dentin after simulated indirect pulp-capping have been published. Previous papers have reported ion diffusion through root dentin from calcium hydroxide [16][17], from canal dressing in simulated external root resorption [18][19][20] and from ProRoot MTA [21][22]. Others have reported pH changes at the surface of root dentin or in the surrounding medium after root canal dressing [23].

In the proposed model, the superficial pulpal layer of dentin on the pulp side was removed in order to obtain a standardized RDT having a maximum permeability [24][25] (with open tubules at both their occlusal and pulp surfaces), to eliminate residual pulp tissue, dentinal plug and tissue proteins (non-collagenous protein and phosphoproteins), and to reduce tubule occlusion by removing rod-like sclerotic casts/mineral deposits in the possible sclerotic/reactionary dentin.

Moreover, an EDTA chelating agent was used to remove the smear layer (cutting debris, intratubular material, mineral deposits, collagen fibrils, proteoglycan linings, bacteria, etc.) and obtain a standardized fully permeable dentin thickness [26][27].

EDTA solutions have been advocated for the effective removal of inorganic as well as organic smear layers. The demineralizing properties of EDTA have been well known for many years [26][27][28][29][30][31]. EDTA solutions allow the removal of water-soluble phosphoproteins and non-collagenous protein, favoring the elimination of residual soft tissues and radicular pulp tissue [31][32].

EDTA is not conventionally used in indirect pulp-capping, despite the fact that the smear layer contains bacteria, their products and necrotic tissue which may act as substrates for bacterial survival and proliferation. Bacteria can deeply penetrate into dentinal tubules and multiply, with the potential to turn them into bacterial reservoirs.

The production of a smear layer on dentin during restorative procedures establishes a protective diffusion barrier [24].

In deep cavities with thin remaining dentin thickness (1.0 mm), the released ions from a pulp-capping material can flow through the dentinal tubules to penetrate the pulp, inducing pulp cell activation and dentin bridge formation.

The removal of the smear layer by EDTA may improve dentin perfusion and increase the positive effect of biocompatible biointeractive materials. For this reason, the samples were treated with EDTA to remove the collagen-depleted apatite phase [33] and allow the migration of dissociated free Ca^2+^ and OH ions into dentinal tubules.

Treatment with EDTA to remove the smear layer has been performed in different previous studies of ion diffusion through dentin in simulated external root resorption defects [21][22] and in simulated canal dressing procedures [20][34][35][36].

In clinical situations, the low pH created by possible residual bacteria in RDT may favor the dissolution of apatite and the smear layer and also trigger the diffusion of ions through the dentinal tubules.

Under in vivo conditions, the tubules are filled with calcium and phosphorous-rich dentinal/tubular fluid at a concentration level near the saturation point. The tubular fluid may reach saturation due to the dissolution of the smear layer by EDTA and the diffusion of Ca^2+^ and OH^-^ ions from the pulp-capping materials. Therefore, calcium phosphate and apatite may likely precipitate inside tubules and on tubular dentin.

In fact, it has been demonstrated that Ca(OH)_2_ is effective at reducing the permeability of both smeared (with a smear layer) and acid-etched dentin in vitro [37][38]. Moreover, calcium-silicate MTA cements trigger the formation of calcium-phosphate crystals [7][8][39][40] inside dentinal tubules, reducing dentin permeability [41]. Both Ca(OH)_2_ and MTA act through the combination of a high concentration of ionized calcium and high pH to produce a local aggregation of calcium phosphates.

The absence of calcium release by the negative controls demonstrated the effectiveness of the applied nail varnish to isolate/waterproof the tooth tissues. The lack of calcium ions detected in the deionized control water demonstrated that no calcium originated from the container itself. Similarly, the fact that no calcium ions derived from the positive controls demonstrated that the immersion in 17% EDTA for 3 minutes effectively removed the smear layer. Actually, the dentin surfaces of the RDT were treated with EDTA to remove the smear layer and any possible source of calcium ions, and to ensure the patency of the dentinal tubules [24][42] in order to standardize the permeability of the samples.

Thus it can be assumed that all Ca^2+^ ions detected in the soaking water derived from the Ca(OH)_2_ of the pulp-capping material and there was no other source of calcium.

Similarly, the release of OH- ions corresponding to an evident increase in the pH of the soaking water was detected only in the medium conditioned by the samples and not in the negative or positive controls.

No statistical analysis and no comments on the release data by the different calcium hydroxide materials are reported in this paper, as the aim was to verify/check and confirm the reliability of the proposed experimental set-up.

All manufacturers of the current pulp-capping Ca(OH)_2_-containing materials have stated the formation of new dentin is related to the ionic dissociation of Ca^2+^ and OH^-^ ions in the presence of fluids [43][44]. However, in indirect pulp-capping the dissociated ions must diffuse into the tubules through dentin migrating to the pulp chamber to fulfill/perform their biological activity.

For this reason the present study was intended to design a simple reliable method to test the dynamics of pulpward flux of Ca^2+^ and OH^-^ ions through dentin after indirect pulp-capping in extracted molar teeth.

To test the validity of the proposed method in the present study, well known chemically distinct pulp-capping materials were used: a non-setting Ca(OH)_2_- based material (Pulpdent paste) present on the market since 1947 [3][45], a self-setting Ca(OH)_2_- based material (Dycal)^1^ available for more than 50 years [46], and a hydraulic water-based Ca(OH)_2_-producing material (ProRoot MTA) introduced in 1995 [47].

Pulpdent paste is a non-setting pre-mixed calcium hydroxide aqueous methylcellulose pulpal dressing composed of calcium hydroxide (42%), an aqueous suspension of methylcellulose and barium sulfate, which was designed by the Dr. H. Berk in 1947 [4][45] and marketed at that time by Rower Dental Manufacturing Company.

Dycal is a self-setting sulphonamide disalicilate containing radio-opaque calcium hydroxide, a plasticizer (sulphonamide) and a setting activator (butylene glycol disalicilate), patented in 1962 [46]. The catalytic paste provides Ca^2+^ and OH- ions while the base paste contains phenolic esters of sulphonamide. The self-setting reaction occurs through an acid-base reaction.

ProRoot MTA is a hydraulic water-based calcium-silicate cement [47]^2, 3^, which is able to form a sticky calcium-silicate-hydrate (CSH) gel and produce calcium hydroxide during its hydration and setting processes [7][8][9][10][48].

In the present study, a simulated pulpal intrapulpal pressure of 0.29 KPa produced by the soaking water in the cylindrical containers (3 cm H_2_O) was used. Normal pulp has a pressure of 1.5 KPa (15 cm H_2_O) and inflamed pulp of 3.5 KPa (36 cm H_2_O) [49]. A low pressure was selected to avoid excessive dilution of the released ions and allow their measurement by the ion-selective probes. A low intrapulpal pressure favors the pulpward movement of ions through the dentinal tubules to the pulp, whilst the ionic dissolution/dissociation from the materials is certainly reduced.

The presence of positive hydrostatic pulpal pressure does not prevent pulpward movement through dentin of calcium and hydroxyl ions leached from pulp-capping agents in in vitro indirect pulp-capping on 1.8±0.2 mm RDT [49]. In fact, in the model proposed in this study, the pulpward movement from the pulp capping materials to the pulpal chamber was permitted despite the presence of a positive hydrostatic pressure.

Levels of intrapulpal pressure can range from 1.5 KPa in normal pulp, to 3.5 KPa in inflamed pulp, and the presence of exudates and dentinal fluid may trigger and maintain ionic dissociation from the pulp-capping material. However, in clinical situations the smear layer is not removed before the indirect pulp-capping, so the pulpward ion diffusion is likely to be reduced.

Moreover, the release of ions from a pulp-capping material and their diffusion is further encouraged by inflamed pulp, since the presence of inflamed pulp is associated with an increase in the pulpal pressure and temperature, with expansion of dentin tubular diameters and reduction of the viscosity of the intratubular fluid resulting [50]. The intratubular fluid movement is affected by the condition of the pulpal tissue. The presence of dentin irritation and/or pulp inflammation can greatly reduce dentin permeability, since plasma proteins leaking from the underlying pulpal vessels can permeate the tubules and be adsorbed to the tubule walls or physically trapped [37].

Fick's law of diffusion describes the diffusive flux of substances, where the driving force is the concentration gradient in relation to the diffusion length, temperature, viscosity of the fluid and size of the particles. The diffusion of chemicals through dentin is closely related to the concentration and molecular size of the chemicals, and with tubule density, diameter and length.

In the proposed model, the pulp flux through dentin of free Ca^2+^ and OH^-^ ions provided by the test materials is likely ascribable, and in relation to, the diffusive permeation across dentin and with the osmotic activity of the materials.

However, it must be pointed out that further phenomena are involved and must be considered in in vivo conditions, such as convective fluid movement, the interaction between the outward convective fluid flux and the inward diffusive flux of molecules, pulpal blood flow and protein permeation and the intratubular material (such as mineral deposits, collagen fibrils, proteoglycan linings, bacteria, etc.). Moreover, the buffering capacity of dentin for alkalis (by displacing phosphate ions) likely contributes to reducing the diffusion and permeation of OH^-^ ions through dentin, as well as the precipitation of calcium phosphate within the dentinal tubules occurring when the tubular fluid becomes saturated with Ca^2+^ and OH^-^ ions released from the pulp-capping materials.

A screening of widely used commercial pulp-capping materials involving a hundred teeth is in progress and is giving reliable reproducible results.

## Conclusions

A simple and reliable method has been designed for the in vitro chemical and physical testing of commercial calcium hydroxide materials.

The coronal RDT system was shown to be reliable for quantitatively measuring the diffusion rates of water-soluble ions derived from the ionic dissolution of different Ca(OH)_2_-containing and Ca(OH)_2_-producing materials for pulp-capping.

## Appendix

### 1

Dycal®, inventor Trademark registration by Dentsply International Inc., “Calcium hydroxide dental cement material” trademark serial number patent 72101309. 1960

### 2

MTA®, inventor Trademark filling (2/5/1996) by Tulsa Dental Products L.L.C., “Dental cement”, serial n. 75053657; abandoned/failure in 1997.1996.

### 3

Proroot®. Trademark registration by Dentsply International Inc., “Dental compounds used in restorative and endodontic procedures” trademark serial n. 75896452. 2000.
